# Transactions of the Medical Association of Pennsylvania

**Published:** 1875-12

**Authors:** 


### Bibliographical.

---

Transactions of the Medical Association of Pennsylvania, held at Pottsville, Pa., June, 1875. Twenty-Sixth Annual Session; pp. 393.

The address of the President, Dr. W. L. Atlee, of Philadelphia, on "Old Physic and Young Physic," contains many ideas of practical interest. Also, the address on medicine, by William Pepper, A.M., M.D., is elaborate and well prepared. The Transactions are filled up with reports from the various county medical societies composing the State association, and are generally well prepared.

